# Transcription Regulators and Membraneless Organelles Challenges to Investigate Them

**DOI:** 10.3390/ijms222312758

**Published:** 2021-11-25

**Authors:** Katarzyna Sołtys, Andrzej Ożyhar

**Affiliations:** Department of Biochemistry, Molecular Biology and Biotechnology, Faculty of Chemistry, Wroclaw University of Science and Technology, Wybrzeże Wyspiańskiego 27, 50-370 Wroclaw, Poland; andrzej.ozyhar@pwr.edu.pl

**Keywords:** liquid–liquid phase separation, membraneless organelles, transcription, nuclear receptors

## Abstract

Eukaryotic cells are composed of different bio-macromolecules that are divided into compartments called organelles providing optimal microenvironments for many cellular processes. A specific type of organelles is membraneless organelles. They are formed via a process called liquid–liquid phase separation that is driven by weak multivalent interactions between particular bio-macromolecules. In this review, we gather crucial information regarding different classes of transcription regulators with the propensity to undergo liquid–liquid phase separation and stress the role of intrinsically disordered regions in this phenomenon. We also discuss recently developed experimental systems for studying formation and properties of membraneless organelles.

## 1. Introduction

To maintain the proper function of the cell, its interior is divided into many compartments called organelles. These functional units contain many bio-macromolecular components (e.g., proteins and nucleic acids) and ensure proper functioning of the cell. Recent research concentrates on particular group of organelles i.e., membraneless organelles (MLOs), also called bio-macromolecular condensates, droplets, granules, foci, or bodies [1]. These structures may occur either in the cell nucleus (e.g., nucleoli, Cajal bodies, and promyelocytic leukemia nuclear bodies (PML-NBs)) or in the cytoplasm (e.g., stress granules (SGs) and processing (P) bodies) [1,2].

MLOs are formed via a spontaneous process stimulated by physiochemical changes in the cell environment. This process, termed liquid–liquid phase separation (LLPS), has been well known for years in polymer chemistry, but recent findings indicate that LLPS is also possible in biological systems [3]. LLPS can be driven by a variety of weak, multivalent interactions [4]. Over the years, a set of criteria defining MLOs have been proposed. Among the most important are spherical shape, ability to fuse together, dynamics, and manner of assembly, regardless of differences in composition, location, and function [3,5], as well as, in some cases, sensitivity to 1,6-hexanediol treatment [6].

Many MLOs possess liquid-like properties [1]. They are highly dynamic and rapidly exchange components with the surroundings. Additionally, the formation of such bio-macromolecular condensates can be easily reversed. However, their properties and organization can change over time [7]. This process is referred to as molecular aging or maturation. The variations in biophysical properties of MLOs have important functional implications. Different environmental factors might lead to functional (e.g., hydrogels formed by nuclear pore complexes (NPCs)) [8] or pathological phase transitions (e.g., solid irreversible structures underlying neurodegeneration) [9,10].

The properties of MLOs allow for a wide range of cell functions. One of them concerns the role of MLOs in transcription [11,12,13,14,15,16]. There are some nuclear MLOs that appear to be involved in different aspects of gene expression regulation. It is already known that the important elements in the formation of such condensates are both the nucleic acids (DNA, RNA) and the interacting proteins [12]. Additionally, it has been shown that the transcription regulators (TRs) often possess the intrinsically disordered regions (IDRs) that are responsible for phase separation [17,18]. However, the dynamics of transcriptional machinery and lack of appropriate tools for MLOs investigation are major challenges for the future study.

There is a strong connection between MLOs, their components, and diseases. Abnormal LLPS lead to progressive loss of the MLOs organization and disfunction. The amyloid beta (Aβ) and tau aggregates are found to be linked with Alzheimer’s disease, the transactive response DNA binding protein 43 (TDP-43) and FET proteins (FUS (Fused in liposarcoma), EWS (Ewing Sarcoma), and TAF15 (TATA binding associated factor 15)) aggregates with amyotrophic lateral sclerosis (ALS) and frontotemporal dementia (FTD) [19]. All above-mentioned proteins undergo LLPS. Additionally, dysregulation of MLOs may be linked to tumor diseases. The PML-NBs are involved in transcriptional regulation and DNA repair but also form in response to viral infection and tumor suppression [20,21].

The review gathers crucial information regarding MLOs formation and their biological implications and highlights the importance of their proper functioning in transcription. We also discuss the challenges in investigating MLOs.

## 2. Driving Forces and Important Role of Inherently Disordered Regions in Liquid-Liquid Phase Separation

**Figure 1:**
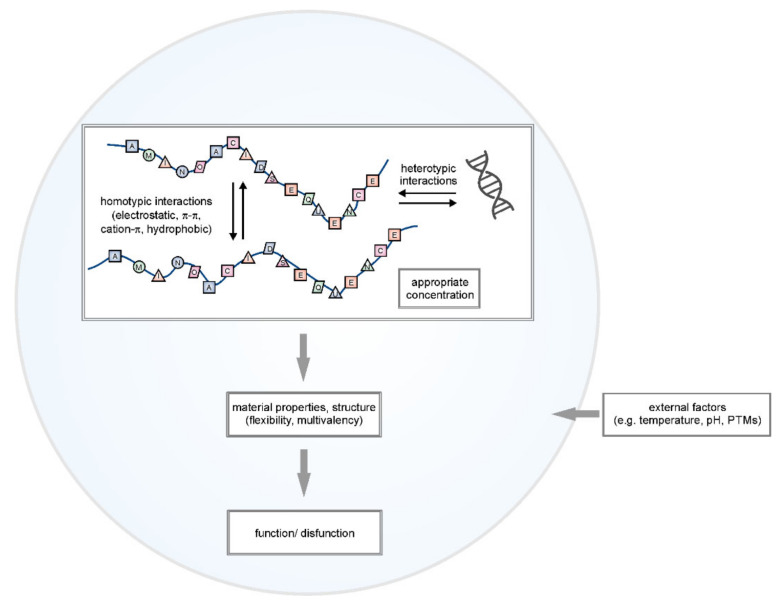
LLPS from amino acid sequence to function. The amino acid composition and the overall sequence patterns determine the interactions, material properties, and structure of protein that encodes the ability to undergo liquid–liquid phase separation. Homo- and heterotypic interactions are depicted. The important regulatory factor is the environment. All these elements define the function or disfunction of particular MLOs (see text for details).

Bio-macromolecules (e.g., proteins, nucleic acids, lipids, and polysaccharides) can interact with each other and organize into a dynamic, highly complex network, which in particular cases can form biomolecular condensates (Figure 1). The formation of MLOs is maintained mostly via multivalent molecules. They are characterized by the presence of multiple regions that enable the contact between different molecules (intermolecular interactions) or within the same molecule (intramolecular interactions) that are important for LLPS. Multiple weak interactions control the partitioning of molecules into two isolated phases: the dense phase (bio-macromolecules-enriched) or the dilute phase (where the local concentration of bio-macromolecules is low) [22]. MLOs’ formation is a concentration-dependent process. Bio-macromolecular condensates contain components that are spatially enriched relative to the surrounding environment. (e.g., responsible for the integrity of MLOs) [23,24].

The interaction between bio-macromolecules in condensates can have a homo- or heterotypic character. Homotypic interactions are those between the same type of molecule. In the opposite are heterotypic interactions, which include different types of molecules (e.g., protein and RNA). Irrespective of the bio-macromolecules themselves, different types of interactions can affect LLPS and MLOs formation. The importance of electrostatic interactions in LLPS have been reported from a number of studies [25,26]. They are classified as long-range interactions and occur between oppositely charged residues. Electrostatic interactions are very sensitive to changes in ionic strength, temperature, or posttranslational modifications (PTMs) [27]. All of these factors enable efficient regulation of MLOs formation. Electrostatic interactions are important, e.g., for DEAD-box helicase 4 (Ddx4) condensates formation. Biophysical studies on the N-terminal region of Ddx4, a primary component of nuage granules [28], indicated that this region is responsible for LLPS of Ddx4 both in vitro and in cell [29]. The characteristic feature of the N-terminal region of Ddx4 is the specific placement of charged amino acid residues, which are arranged into clustered blocks of net positive and negative charge. Condensate formation of Ddx4 is sensitive to salt concentration and temperature as well as methylation of R residues that leads to dissolve the condensates. Electrostatic interactions have been explored in the context of many proteins, for which LLPS showed a similar salt dependence as for Ddx4, although charge-arrangement features were not observed for all of them. Except the methylation of R residues mentioned earlier, phosphorylation is also an important PTM, as it modifies the net charge of proteins and modulates electrostatic interactions. Depending on the protein context, the substitution of hydroxy functional group of S, T, and Y residues with a negatively charged phosphate group can either promote or disrupt LLPS [27,30,31].

Another type of interaction that plays an important role in LLPS is short-range interactions such as cation–π (which occurs between the positively charged residues (e.g., R, K) and the aromatic residues (e.g., Y, F)) [32], π–π (aromatic residues) [33], and dipole–dipole (prion-like sequence/regions) [3,34]. Each of these interactions play a vital role in the formation of particular MLOs (e.g., cation–π interactions are very important for ribonucleoprotein (RNP) granule formation). The formation of bio-macromolecular condensates can be also driven by hydrophobic interactions [35]. One well-described example is elastin, in which hydrophobic patches are required for phase transition and subsequent filament assembly [36]. Hydrophobic interactions are also important for LLPS of model proteins, e.g., FUS, TDP-43, and Annexin A11, to investigate the relationship between LLPS and aggregation.

In many cases, the combination of different types of molecular interactions can also drive LLPS. Ddx4 undergoes not only LLPS mediated by the above-discussed electrostatic interactions but also cation–π interactions [29]. Mutation F to A residues prevent the phase transition of Ddx4. The Tau protein also undergoes LLPS driven by electrostatic interactions either itself or in the presence of RNA [37,38]. On the other hand, the LLPS of tau can be also driven by hydrophobic interactions in the presence of high salt concentration [39].

Different environmental factors may affect the dynamic of MLOs formation (Figure 1). The most important include the concentration of proteins, the presence of nucleic acids, PTMs, temperature, pH, and salt concentration [40]. Their changes may affect protein solubility, affinities between bio-macromolecules, and phase behavior. MLOs form when the concentration of key elements crosses its critical saturation limit. Increased temperature can increase the thermal motion of molecules and lead to their dissociation from the complex. It can also reduce the binding of the solvent molecules and increase the direct contact between macromolecules. The pH change in solution affects LLPS by changing long-range interactions, such as electrostatic. The temperature and pH changes are commonly associated with cellular stress that leads to the formation of SGs [41].

MLOs formation can also be regulated by PTMs [27]. Phosphorylation of S/T or methylation of R residues change the properties of amino acids, alter interaction strength, and affect phase behavior, either promoting or repressing. S/T phosphorylation of the Tau protein promotes LLPS and SGs formation by increasing electrostatic interactions [38,42], where S/T phosphorylation of FUS introduces electrostatic repulsion and prevents phase transition [43]. Y phosphorylation may also play a critical role in regulating LLPS and MLOs formation. The heterogeneous nuclear ribonucleoprotein A2 (hnRNPA2), the component of hnRNPA2 transport granules, is a known target of Y phosphorylation [44] that regulates the release of mRNA from hnRNPA2 granules for translation in cells [45]. Veronica Ryan and coworkers showed that Y phosphorylation of hnRNPA2 reduces its phase separation [46]. It also prevents partitioning of other proteins (e.g., heterogeneous nuclear ribonucleoprotein F (hnRNPF) and cytoskeleton-associated protein 5 (CKAP5)) of hnRNPA2-containing transport granules into hnRNPA2 droplets. Additionally, Y phosphorylation decreases the aggregation of hnRNPA2 disease variants.

Salt concentration is another important factor that influences the way bio-macromolecules interact with one another and triggers LLPS [35]. Proteins can be divided into two groups—the first forms condensates under low salt concentrations, and the second undergoes LLPS under high salt conditions. Recently, it was shown that several proteins (e.g., FUS, TDP-43, bromodomain-containing protein 4 (Brd4), sex-determining region Y-box 2 (Sox2), and Annexin A11) can exhibit reentrant phase separation behavior [35]. These proteins undergo LLPS via homotypic multivalent interactions at low salt concentrations. They can also undergo LLPS at high salt concentrations, what was called reentering into a phase-separated regime. In the high salt regime, the condensates are sustained predominantly by hydrophobic and non-ionic interactions. It was found that the molecular interactions, stabilizing the condensates in the low- and high-salt regime, are fundamentally different. The hydrophobic and electrostatic interactions are both important at low salt concentrations, whereas LLPS is mainly driven by hydrophobic and nonionic interactions at high salt concentrations. These results emphasize that phase separation is strongly dependent on different environmental factors.

Another important aspect of MLOs is the amino acid composition and sequence pattern of proteins that can undergo LLPS (Figure 1). Many MLOs are enriched with intrinsically disordered proteins (IDPs) or proteins that contain intrinsically disordered regions (IDRs) [17,47]. IDPs/IDRs are enriched in amino acid residues such as A, R, G, Q, S, P, E, and K (disorder-promoting) and depleted of amino acid residues such as W, Y, F, V, I, L, C, and N (order-promoting). Moreover, IDPs/IDRs can contain the characteristic pattern of amino acid residues with little diversity in amino acid composition, the so-called low complexity sequences (LCSs) [48]. This array of amino acids allows the formation of specific bonds that promote LLPS [49,50]. IDRs with specific sequence features seem to enrich different biomolecular condensates [51]. The R-rich IDRs are important for forming nucleoli, and the S/R-rich IDRs are critical in forming nuclear speckles. P-bodies contain proteins with Q/N-rich regions [52], while the hydrogel-like structure of the nuclear pore complexes (NPCs) contains proteins with an FG-rich sequence [8].

IDPs/IDRs lack a fixed ordered three-dimensional structure and, therefore, they are characterized by high flexibility. Their structural plasticity enables them to adopt different conformations. This conformational flexibility of IDPs/IDRs provides a large interaction surface with high-specificity and low-affinity binding properties that are ideally suited for the transient reversible interactions involved in LLPS. Consequently, IDPs/IDRs might interact with multiple partners (multivalent interactions); thus, a whole network of non-covalent interactions can arise [53]. IDPs/IDRs are engaged in diversity of biological functions, e.g., signal transductions and regulations, where they form complex interaction networks, often involving many partners [54]. Not only IDPs/IDRs take part in formation of MLOs. In some cases, interactions between repeated, folded domains of proteins are required for the formation of assemblies [55]. However, IDPs/IDRs are the vast majority.

IDPs/IDRs can adopt many different structures in vivo depending on the cell conditions. They often contain short functional elements called short linear motifs (SLiMs), which mediate protein–protein interactions [56]. These motifs are often sites of PTMs [57,58]. Several studies indicate that PTMs have a strong influence on the regulation of LLPS [27,31]. These features make IDPs/IDRs well-suited for MLOs formation.

## 3. Structure and Roles of Bio-Macromolecular Condensates in the Cell

A cell may be seen as a network of many different MLOs [59]. In response to changes in the cell environment, they rapidly assemble or disassemble. Important element of this network is communication between condensates that can exchange their constituents with the surrounding. Bio-macromolecular condensates contain many different components, for which a particular role can be assigned. Protein components of MLOs can be classified into four types: scaffolds (drivers), co-scaffolds (co-drivers), clients, and regulators [60,61]. Scaffolds are essential constituents of each condensate and are responsible for its integrity. This role was assigned to spindle-defective protein 5 (SPD-5), which is sufficient for the formation of centrosomes in *Caenorhabditis elegans* [62]. Another example is promyelocytic leukemia (PML) protein, which is essential for PML-NBs formation [63]. Co-scaffold is a component that needs another co-scaffold to phase separate (e.g., RNP and RNA) [52]. In contrast, clients are dispensable components for MLOs assembly. They reside in the MLOs only under certain conditions [64] and exchange with the surroundings much more rapidly than scaffolds/co-scaffolds [65]. The last type consists of proteins called regulators, which promote LLPS but are not located in the condensates (e.g., modifying enzymes) [61]. Despite the difference in composition, location, and function, a set of criteria defining MLOs has been proposed. The most important are spherical shape, manner of assembly, and dynamic components that undergo external exchange with the surrounding [5]. Further characteristics are deformation in response to applied force [66], concentration-dependent size scaling [67], and, in the case of hydrophobic interaction-driven condensates, sensitivity to 1,6-hexanediol [6].

The intramolecular and intermolecular interactions between bio-macromolecules within MLOs can lead to formation condensed phases that are characterized by different states and material properties (Figure 1). These assemblies can be liquid, colloid, or solid-like forms (e.g., gels, crystals, glass, or filaments) [1,68]. In liquid forms, bio-macromolecules are highly dynamic. They can form assemblies that are sensitive to many factors (e.g., components concentration, temperature, and ionic strength). They can also constantly exchange elements with the surrounding [1]. The enhancement of the strength or change of the type of interactions between bio-macromolecules in the assemblies can cause the liquid-to-solid phase transition, where the molecules are arranged in a more ordered structure. This process is called maturation or molecular aging and results in the loss of flexibility of components [7]. MLOs can mature into a gel or glass form. However, depending on the cell conditions, they can also mature into more solid-like structures. There are many biological implications of these phase transitions. Exchange components between the nuclei and cytoplasm proceeds through the NPCs. It was shown that NPCs have sieve-like structures that are created through reversible cross-linking between FG-rich nucleoporin repeats that can form elastic and reversible hydrogels [8]. Another example can be inclusion bodies, MLOs that serve to concentrate the viral RNA replication machinery of measles virus (MeV). These inclusion bodies can change from liquid to gel-like structures as infection progresses [69]. However, the best-described examples are groups of RNA-protein (RNP) granules, P bodies, and SGs, which can adopt different material states depending on the cell conditions and organism. Their physical properties range from liquid-like in mammalian cells to solid-like in yeast [70]. Their properties are also compatible with their functions. P bodies are active compartments involved in RNA metabolism. Their liquid-like form allows for the continuous flow of molecules and components rearrangement. On the other hand, SGs exhibit characteristic properties of amorphous aggregates. They store and inactivate proteins and RNAs by removing them from the cytoplasm [71,72].

Bio-macromolecules undergo not only functional but also pathological phase transitions [50]. Maturation can coincide with formation of solid irreversible structures. Alternation of the material properties represents a common principle underlying neurodegeneration. Many proteins (e.g., FUS, TDP-43, TIA1, tau, α-synuclein) associated with Parkinson’s disease or amyotrophic lateral sclerosis (ALS) also undergo LLPS and are constituents of different types of MLOs [9,10,73,74,75]. It suggests that the liquid-to-solid phase transition can be enhanced within the liquid phase. Important factors in this transition are time, conditions, and components of MLOs.

Bio-macromolecule condensate formation can have many functional aspects. LLPS has been shown to maintain spatiotemporal intracellular organization, tune and accelerate biochemical reactions, act as biomolecular filters, modulate signal transduction, regulate nucleic acid metabolism, sequester and release specific components, protect biomolecules from damage, and buffer cellular noise [2,24,40,76,77,78]. Many proteins that undergo LLPS have already been identified. Additionally, some of them have been assigned to particular known MLOs. A few databases, covering different aspects of phase separation of bio-macromolecules, have been developed [79] (see Chapter 5). However, many condensates are still waiting for the identification. A lot of them may be not large enough to be easily identified. Moreover, some condensates can form only on specific stimuli. Thus, the biggest challenges are identification MLOs, their components, and designation biological functions.

## 4. Transcription Regulators and Liquid–Liquid Phase Separation

**Figure 2:**
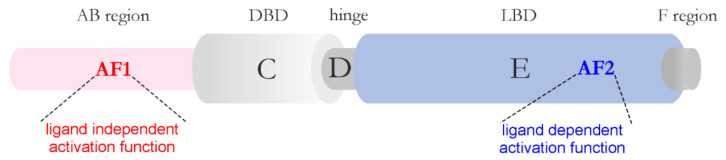
Structural organization of nuclear receptors. Nuclear receptors exhibit a modular structure with different regions from A to F. Some of them correspond to autonomous functional domains: the DNA-binding domain (DBD) and ligand-binding domain (LBD). Nuclear receptors have two activation functions (AFs), the ligand-independent (AF1) function, which is localized in AB region, and the ligand-dependent function (AF2), which resides in LBD.

In recent years, the occurrence of bio-macromolecular condensates in the nucleus has become the subject of detailed genetic, biochemical, and structural studies [80]. It has been suggested that nuclear condensate formation might be important for the regulation of various aspects of gene expression, as multiple factors responsible for the process undergo LLPS. One of the essential regulators of eukaryotic gene expression is transcription factors (TFs). Nuclear receptors (NRs) are one of the largest family of eukaryotic TFs that not only bind DNA but also are ligand-dependent. NRs are multivalent molecules. Most of them dimerize, which helps to establish multivalent interactions. Additionally, they interact with many transcriptional coregulators. Two regions, an AB region (N-terminal domain, NTD) and an E region (ligand binding domain, LBD), which harbor the activation functions AF1 and AF2, respectively, are important for these interactions (Figure 2). The AF2 is strictly ligand-dependent, whereas AF1 is ligand-independent. Recent reports linked the regions that contain AFs to the LLPS phenomenon [81]. In steroid NR family representatives, the androgen receptor (AR), the estrogen receptor (ER), and the glucocorticoid receptor (GR), LBD (AF2) seems essential for this process [81,82,83]. It was found that, in a cellular model of prostate cancer, only full-length AR could phase separate on its own [84], where its splice variant lacking LBD, AR-v7, did not undergo LLPS [83]. This indicates that a cooperative interaction between NTD and LBD of AR is important for the phase separation of this receptor [83]. Additionally, in vitro analysis of AR NTD showed that it underwent LLPS at 100 μM concentration and the presence of the tumor suppressor speckle-type POZ protein (SPOP) lowered the concentration at which NTD of AR could phase separate [85]. In vitro analysis of individual regions of AR showed that in the presence of RNA and DNA DBD could also undergo LLPS [83]. The process was downregulated by AF1 located in AR NTD. Analysis of individual regions of GR showed that the AB region (AF1) was not responsible for formation of the GR condensates but only stabilized them [82]. Additionally, the AR, ER, and GR condensates were observed in the presence of the Mediator Complex subunit 1 (MED1). Upon ligand stimulation (estrogen), the incorporation of the ER into the MED1 droplets was enhanced [81]. In the case of GR, the formation of condensates required interaction of the receptor with certain chromatin regions within the nucleus. In the presence of the ligand, AR and GR, and also other steroid receptors, are translocated to the nucleus, where they form transcriptionally active foci. It was shown that AR and GR foci exhibit properties of MLOs (e.g., ability to fuse, dynamics, and sensitivity to 1,6-hexanodiol/1,7-heptanediol) [82,84]. The nature and functional relevance of steroid receptors’ foci were reviewed extensively in [86].

The non-steroid NRs representative, the retinoid X receptor (*h*RXRγ), is also able to form characteristic foci in the nucleus of COS cells in the presence and absence of ligand (data not shown). Some fraction of RXR is also present in the cytoplasm and plays an important role in translocation of other NRs to the nucleus [87]. The point mutant of *h*RXRγ that has cytoplasmic localization can form foci only after ligand stimulation (data not shown). Probably, these cytoplasmic foci of RXR might allow the formation of a temporarily reservoir for other NRs. However, the real function of these foci is unknown.

An intrinsically disordered AB region of *h*RXRγ seems to be responsible for LLPS of this receptor. It was shown that this region forms condensates in vitro in the absence of other proteins [88]. These condensates are able to incorporate the remaining fragment of the receptor into the droplets. Although the AB region of *h*RXRγ shows the characteristics of IDPs/IDRs, the condensate formation is driven by hydrophobic interactions, which is rarely described in the case of IDRs, as IDRs do not usually have many hydrophobic residues [89]. Among NRs, the AB region shares little sequence homology. It is characterized by a variable length and sequence in the different family members of NRs and often exhibits properties of IDRs under physiological conditions [90]. The AB region of NRs contains AF, which is an important determinant of the subtype-, cell-type-, and gene-specific functions of NRs [91]. The difference in AB regions can impact propensity for droplet formation between NRs and can be critical for the modulation of the transcriptional activation of target genes. It was suggested that AFs are responsible for the phase separation of TFs [81]. The composition, intrinsic disorder, and ability to interact with many partners (multivalency) of AFs make them perfect candidates for MLO formation and regulation of transcriptional activities [18]. Additionally, TFs contain DBDs that target specific genomic loci. Thus, TFs might function as nucleation centers or scaffolds of MLOs [92].

The propensity for LLPS is characteristic not only for NRs but also for other TFs such as MYC, p53, NANOG, SOX2, and GATA2 [81]. However, not all TFs have the propensity to induce LLPS. For example, octamer-binding transcription factor 4 (OCT4) does not undergo LLPS alone but is incorporated into condensates formed by MED1 [81]. Mediator complex (MED) seems to be another important element that is involved in MLOs formation. In mammals, MED is a large size complex composed of about 30 subunits that can be exchanged [93]. MED is not only dynamic in its subunit composition, but its particular subunits are also intrinsically disordered [94]. The nature of MED facilitates communication and diverse functional interactions with TFs bound to enhancer-promoter regions. Thus, MED may also serve as a scaffold around which other components of transcriptional machinery assembly (e.g., polymerase II RNA, pre-initiation complex (PIC)) and maintain the integrity of the condensates. This reveals completely new structural or functional roles of MED. However, there are many questions to be answered. Today, particular components of transcriptional machinery that undergo LLPS have been identified. However, it is not known if TFs form independent condensates that are fused with MED’s condensates or they only target specific genomic loci where condensates will appear and form MLOs, as the endogenous concentration of TFs may be not sufficient to form condensates in the cell.

The important element of transcriptional control is the subcellular distribution of transcriptional machinery. Formation of MLOs could provide an easy way for localization of proteins and nucleic acids in a spatial and temporal manner [95,96]. Additionally, such condensates are selective, admitting only specific components and excluding others. LLPS strongly depends on the local concentration of critical components. Using live-cell super-resolution imaging methods, Won-Ki Cho and coworkers showed in mouse embryonic stem cells (mESCs) that endogenous MED and RNA polymerase II (RNA pol II) form condensates that can colocalize and have properties of MLOs [97]. The model was proposed in which condensates of MED are recruited at a given locus by TFs and interact with condensates formed by RNA pol II to promote gene activation. Shasha Chong and coworkers showed that TFs are also an important element of this model. Employing single-molecule imaging, they studied, in living cells, LCS–LCS interactions of a subset of TFs such as EWS/friend leukemia integration 1 transcription factor (FLI1), TAF15, and Sp1 [98]. They showed that the LCS–LCS interactions are dynamic, multivalent, and sequence-specific, which enables forming transient local regions of high TF concentrations. Although LCS–LCS interactions and regions of high TF concentrations were observed at endogenous expression levels, there was no evidence for their phase separation. LLPS of LCSs was detected only after overexpression of TFs. Peng A and Stephanie Weber proposed that condensates can form through at least three distinct mechanisms: (1) binding of proteins to nucleic acid; (2) bridging, where proteins bind to more than one nucleic acid site at a time; or (3) LLPS [99]. Each of them represents different concentration dependence and diffusion across the boundary. It is probable that LCS–LCS interactions for TFs under investigation lead only to regions with local enrichment of TFs that do not undergo LLPS what is related to their concentration.

Another important aspect of transcriptional condensates is DNA sequences. Krishna Shrinivas and coworkers demonstrated that multivalent DNA elements can serve as scaffolds for the phase separation of transcriptional condensates [92]. Low concentrations of TFs and coactivators, which are too low for LLPS, can be sufficient for condensates formation in the presence of specific DNA sequences. It was shown that the affinity, number, or density of TF–DNA interactions have a strong impact on condensate formation. Thus, a large number of binding sites for TFs in DNA sequences might not be accidental but might evolve to concentrate TFs and enable interactions with MED to form MLOs.

Recent studies have suggested that bio-macromolecular condensates form at super-enhancers (SEs) [81,96,97,100,101]. This model was supported inter alia by the ability of BRD4 and MED1, a key component of SEs, to form condensates at sites of SE-driven transcription. SEs’ condensates bring together many TFs and coactivators containing IDRs with propensity to LLPS at specific genomic regions and allow for highly selective transcriptional activation [100]. For formation of transcription condensates, two types of interactions seems to be important—specific interactions between TFs and DNA sequences and transient, multivalent interactions between IDRs that regulate formation or stabilize the condensates.

Many components of transcriptional machinery that undergo LLPS are subjected to reversible PTMs [31]. One of them is the RNA pol II [102,103]. The C-terminal domain (CTD) of RNA pol II is a disordered LCS that might have different phosphorylation pattern depending on the stage of transcription (initiation, elongation, or termination). At transcription initiation, the CTD is unphosphorylated, and it can be incorporated into condensates formed by other components of transcriptional machinery, such as TFs and MED. Phosphorylation of CTD by TFIIH (via its CDK7 kinase subunit) and positive transcription elongation factor b complex (PTEFb), which includes the kinase CDK9, promotes transfer from initiation to elongation condensates [103]. The phosphorylated CTD of RNA pol II can also be incorporated into a condensates formed by splicing factors [104]. These results show that RNA pol II plays the role of a client that can reside in the condensates under certain conditions. They also stress the ability of PTMS to modulate the composition of condensates. Dephosphorylation of the CTD of RNA pol II induces transcription termination [105,106]. Thus, LLPS play the role on each stage of transcription, from initiation to termination.

Many classes of RNA, both coding and non-coding, play an important regulatory role in the phase separation and MLOs formation. Recently, it was shown that the changes of RNA concentration during the transcription process dynamically regulate the behavior of transcriptional condensates [107]. A non-equilibrium feedback control mechanism was proposed. During transcription initiation, there is a low level of short RNAs that stimulate condensate formation. RNA molecules promote condensate formation through electrostatic interactions with proteins [108]. During transcription elongation, a high level of longer RNAs appear, so the negative charges are much higher than the positive charges, which causes the repulsion between the charges and condensate dissolution [107]. In addition, the specific secondary structure of mRNA can also regulate LLPS by influencing interaction between mRNAs and RNA-binding proteins (RBPs) [109]. Another important class of RNA molecules that plays a diverse role in gene expression and regulates LLPS is non-coding RNAs [110]. In general, it is believed that they provide an essential scaffold or platform for RBPs that promotes protein–protein interactions and leads to MLO formation such as nuclear bodies (NBs) [52,110,111,112]. It has been even proposed that this subset of non-coding RNAs should be designated as “architectural RNAs” (arcRNAs) [113]. Recent studies revealed that particular NBs (e.g., deleted in breast cancer 1 (DBC1)—containing NBs) are built using specific arcRNAs that are important for their formation and maintenance [114]. These data clearly indicate the important role of RNA molecules in LLPS and MLOs formation.

RNA molecules can be also involved in the formation of MLOs through their interaction with proteins that contain IDRs. R-loops are three-stranded structures composed of an RNA–DNA hybrid and a displaced strand of DNA [115]. These structures have an important role in many cellular processes concerning DNA replication, repair, and transcription. High R-loop levels can lead to genome instability and chromatin alterations [116]. Many proteins that interact with R-loops have been identified. The C-terminal of Fragile X Protein (FMRP), which exhibits the properties of IDRs, is the predominant R-loop binding site [117]. Additionally, it has the propensity to undergo LLPS alone or in the presence of RNA molecules [118]. The analysis of the R-loop interactome showed that many R-loop processing and signaling proteins contain long IDRs that are highly enriched in LCS [119]. It was suggested that these IDRs could be the predominant sites for interaction with R-loops, as was shown for the C-terminal of FMRP. Additionally, several proteins in the R-loop interactome undergo LLPS. These data suggest that LLPS might be an important aspect of R-loop biology.

Spatial organization of chromatin (both euchromatin and heterochromatin) may also be attributed to LLPS and MLOs formation [120]. Different factors such as, e.g., DNA modifications, DNA-binding proteins, and PTMS of histones, may act through LLPS to affect chromatin organization [121]. One of well-known non-histone chromatin-associated group of proteins is the heterochromatin protein (HP1) family. HP1s take part in chromatin condensation [122], modulation of chromatin dynamics [123], and regulation of transcription [124]. The alterations in the HP1 expression are linked to different types of cancers. Recently, it was shown that the human heterochromatin protein 1α (HP1α), which is the major component of heterochromatin, undergoes LLPS [125]. The process is driven by two IDRs of HP1α—the N-terminal extension (NTE) and the hinge region. Additionally, the phase transition of HP1α is phosphorylation-dependent [125]. In humans, there are three isoforms of HP1 (HP1α, HP1β, and HP1γ), but only HP1α is involved in LLPS. Moreover, the *Drosophila* HP1a also exhibits LLPS in similar conditions, but, unlike human HP1α, it does not require any PTMs [126]. Summarizing, LLPS can lead to chromatin condensation and consequent repression of gene transcription.

Alternation of transcription program can lead to different types of cancers [127]. Dysregulation of the *cis*- (e.g., SEs) and *trans*- (e.g., TFs, coactivators) transcription regulators described earlier that undergo LLPS can result in the aberrant expression of oncogenes and facilitate tumor progression [128]. For example, EWS-FLI, the fusion product of intrinsically disordered amino-terminal domain of EWS and the carboxyl-terminus of FLI1 containing DBD, is the key oncogenic protein in Ewing sarcoma [129]. The EWS-FLI have ability to form condensates that are essential for transcription activation and oncogenic gene expression programs in tumor cells [98]. As already mentioned, the LLPS phenomenon is also present in the viral life cycle. Many viral proteins are enriched in IDRs. Additionally, many of them have the ability to undergo LLPS and form so-called inclusion bodies (viral factories), structures that are associated with viral replication and trafficking of viral components [130]. The nature and functional relevance of a several examples of inclusion bodies were reviewed extensively in [131]. Moreover, two recent studies provide evidence that LLPS during viral replication might be a target for antiviral therapy [132,133]. Thus, MLOs play important regulatory roles in transcription and viral replication and can also constitute new approaches to disease therapy.

## 5. Challenges in the Investigation of the Condensates

LLPS has emerged as a principle of cellular organization. The growing interest in LLPS has led to the development of a few databases: RNA Granule Database [134], PhaSePro [135], Pha-SepDB [136], DrLLPS [137], and LLPSDB [138]. They gather information from the literature about proteins or protein regions with in vivo and in vitro experimental data, associated with LLPS or associated with known MLOs. They also provide a range of information on driving forces of LLPS, conditions for condensate formation, and enable the definition of function and components of particular MLOs. In parallel with databases, several bioinformatic tools (e.g., PLAAC [139], catGRANULE [140], Pscore [33], and PSPer [141]) for predicting proteins with propensity to LLPS have been developed. The description of the algorithms and applications of each tool, their comparison, and their strengths and limitations have been described previously [142,143]. These tools might provide new targets for experimental validation. However, it is important to take note of the type of target protein for correct interpretation of the results as a variety of mechanisms by which phase separation might occur. Recently, to improve existing methods, a new predictor called PSAP was developed [144]. It is based solely on amino acid content of proteins from human proteome that can form liquid condensates in vitro and in vivo. Comparison of these proteins with the rest of the human proteome enables the generation of a list of amino-acid-related features that could discriminate proteins with propensity to LLPS and generate a machine learning algorithm to predict proteome-wide protein phase separation.

Although many components of bio-macromolecular condensates have already been identified, there is still a significant gap between in vitro and in vivo studies. During in vitro studies, it is much easier to control many factors, which do not always reflect real conditions in the cell. There are many models that are designed to mimic specific aspects of condensates in the cell. For example, inert synthetic polymeric molecules such as polyethylene glycol (PEG), Ficoll, and dextran have been used to simulate the densely crowded environment of the cell [145]. However, the behavior of IDRs, that very often drive LLPS, in the presence of molecular crowding agents can be very complex [146]. Additionally, the macromolecular crowding agents might affect protein structure and folding and also impact LLPS [147]. Another element that needs to be considered is the dependence of condensates formation on concentration of some components. The overexpression of protein can lead to the formation of characteristic foci that might not have properties of MLOs [98]. In the cells, an appropriate concentration of proteins with propensity to undergo LLPS needs to be considered to preserve their functional role in MLO formation. Nazanin Farahi and coworkers found that genes coding for proteins that undergo LLPS tend to be dosage-sensitive [61]. This tight regulation prevents harmful changes (increases or decreases) in protein concentration under physiological conditions. Another challenge is the size of particular condensates. Some types of RNP granules are difficult to study because they are small, dynamic, and restricted to specific cell types such as neurons or germ cells [148].

LLPS can be monitored using various methods. There are many techniques for in vitro studies based on measurements of optical density and light microscopy (contrast- or fluorescence-based microscopy). There is much less possibility when it comes to in cell study, which often requires super-resolution microscopy [97,98]. A common technique is fluorescence recovery after photobleaching (FRAP), which enables the monitoring of the diffusion of fluorescent-labeled proteins within a photobleached region and the assessment of macromolecular fluidity within phase-separated condensates [149]. Similar liquid-like properties and concentration-dependent formation were defined for many MLOs [50,66] and became one of criteria defining the new ones. However, using FRAP, appropriate experimental conditions (e.g., the bleach spot size and the ratio of bleach spot to drop size) need to be applied as they can influence obtained results. Nicole Taylor and coworkers prepared the guidelines to determine an appropriate model used to fit FRAP data [150].

An important chemical that enables LLPS investigation is 1,6-hexanediol. This compound is known to disrupt liquid-like condensates by interfering with hydrophobic interactions [108]. Sensitivity to 1,6-hexanediol and also 1,2-pentanediol or 1,2-hexanediol is the characteristic for MLOs, for which contribution of hydrophobic interactions is observed. In the case where LLPS is driven by electrostatic interactions, 1,6-hexanediol has no effect [83]. However, Yuji Itoh and coworkers showed that 1,6-hexanediol removes water molecules around chromatin and locally condenses it. Thus, results should be carefully interpreted when the droplets are associated with chromatin [151]. Both FRAP study and sensitivity to 1,6-hexanediol may not always be sufficient to demonstrate that particular structure represents liquid-phase condensate.

**Figure 3:**
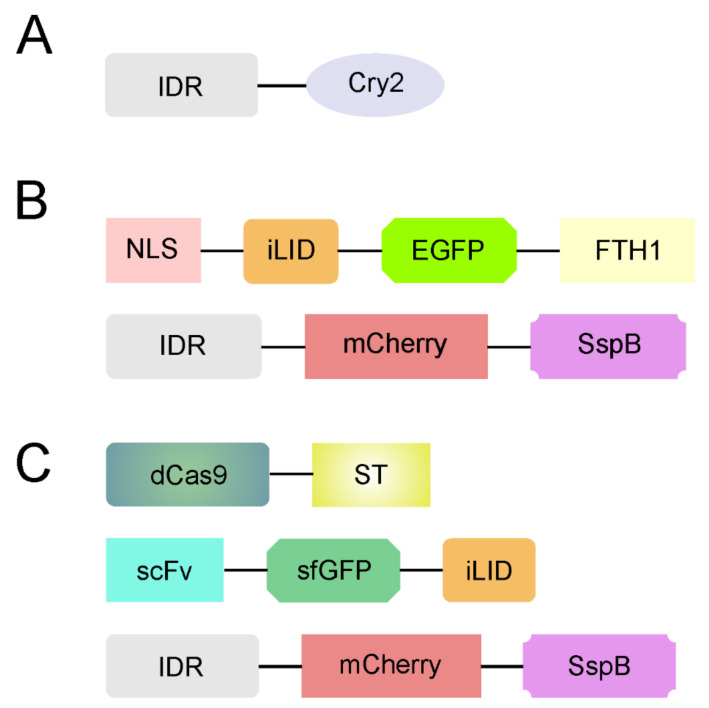
The basic modules of optogenetic tools use to investigate phase separation. Schematic diagram of the (**A**) optoDroplets [112], (**B**) Corelet [114], and the (**C**) CasDrop system [113]. For details, see the text.

Recently, a group of optogenetic tools to investigate LLPS appeared (Figure 3) [152,153,154,155]. optoDroplets is a photo-activated system developed for reversible controlling IDR-driven phase transitions [152]. As most optogenetic tools, optoDroplets is constructed by fusing the photoreceptor (Cry2) to cellular effector molecules (IDRs of FUS, DDX4, and hnRNPA1), the activity of which can subsequently be triggered by light (Figure 3A). Only above a threshold concentration, upon blue light activation, the investigated constructs underwent LLPS, forming spatiotemporally liquid droplets. Thus, the optoDroplets system enabled the observation of the phase transition under physiological conditions.

The core scaffolds to promote droplets (Corelet) are another optogenetic tool that was developed for mapping local and global liquid phase behavior [154]. The basis of this method are two modules (Figure 3B). The first consists of 24 human ferritin-heavy chain (FTH1) protein subunits (“core”) fused to a nuclear localization signal (NLS), EGFP protein, and an improved light-inducible dimer (iLID) domain. The second consists of a sequence under investigation (e.g., IDR of FUS) fused to mCherry tag and SspB. The iLID heterodimerizes with SspB in response to blue light [156], which, in turn, enables IDR-containing liquid droplet formation, even under globally dilute IDR concentrations. Thus, Corelet provides an opportunity to map intracellular phase diagrams.

TFs are often enriched at specific DNA sequences near genes where they may undergo LLPS. The CRISPR-Cas9-based optogenetic platform termed CasDrop was developed to investigate formation of condensates at specific genomic loci in the cell [153]. The modular components of the CasDrop include (1) effector protein Cas9 (dCas9), which can be targeted to any sequence in the mammalian genome using sequence-specific small guide RNAs (sgRNAs) fused to SunTag (ST) [157]; (2) single-chain variable fragment (scFv) antibody, cognate for the ST, fused to super-folder GFP (sfGFP) and iLID; and (3) the sequence under investigation (e.g., IDR of BRD4, FUS, and TAF15) fused to mCherry tag and SspB (Figure 3C). The first two components can self-assemble into a multimeric protein complex that allows for the visualization of seeded sites. The third component provides light-inducible binding scaffolds for recruiting IDRs [153]. The CasDrop system was used to show that IDRs can bring distal genomic loci together to form liquid condensates while mechanically excluding non-specific neighboring genomes.

The propensity to undergo LLPS seems to be a universal property of bio-macromolecules under defined conditions. The in vitro and in cell studies of recent years allow obtaining valuable knowledge about different aspects of phase separation in cell biology. They also led to the development of a few databases and several computational predictors, which might provide new targets for experimental validation. However, further studies are required to fully characterize the biophysical properties of MLO components and mechanism of MLO formation. A lack of appropriate tools to observe LLPS in cells limits the ability to study their role in cell function and disfunction. A major future challenge is having an accurate set of methods for investigating MLOs and demonstrating that a specific high-concentration region of bio-macromolecules is indeed a phase-separated organelle in the context of the cell.

## Data Availability

Not applicable.
